# Klebsiella, a Clever Survivor Presenting As Pyogenic Liver Abscess Years After Travel

**DOI:** 10.7759/cureus.38743

**Published:** 2023-05-08

**Authors:** Joseph P Moran, Guadalupe Osorio, Kindra Holsing, Aneesa Afroze

**Affiliations:** 1 Internal Medicine, MercyOne Des Moines Medical Center, Des Moines, USA; 2 Infectious Disease, MercyOne Des Moines Medical Center, Des Moines, USA

**Keywords:** sepsis, travel-related infection, infection microbiology, pyogenic liver abscesses, klebsiella pneumoniae (kp)

## Abstract

*Klebsiella pneumoniae* is a known cause of pyogenic liver abscess and has an increased prevalence in Southeast Asia. We present two cases of individuals with remote travel history to southeast Asia presenting with fevers, chills, and abdominal pain secondary to pyogenic liver abscess. Neither individual had a comorbid medical condition or history of prior hepato-biliary pathology that would predispose them to bacterial translocation and abscess formation. These patients were both successfully treated with percutaneous drainage and antibiotics. We present these cases to add to the growing body of literature surrounding hyper-mucoid strains of *Klebsiella pneumonia* causing a pyogenic liver abscess.

## Introduction

We present two cases of individuals diagnosed with mucoid *Klebsiella* liver abscess presenting with bacteremia. These cases occurred in the state of Iowa in the United States in 2022. Both patients had a remote history of immigration from Southeast Asia. *Klebsiella pneumoniae* is a well-known nosocomial pathogen that has evolved as a more prevalent cause of pyogenic liver abscess. It is well known to have a higher endemic prevalence in the populations of Southeast Asia but remains rare in the United States [1].

Since 1986, hypervirulent strains of *Klebsiella pneumoniae* causing pyogenic liver abscesses have been documented in the literature [2]. Likewise, a multitude of virulence factors have been associated with these hypervirulent strains of mucoid Klebsiella, including larger mucoid capsule production and increased siderophore expression [3] [4]. The typical presenting symptoms are fevers, chills, and abdominal pain [5]. If not recognized promptly, the hematologic spread of infection may lead to multiple complications.

Case 1 in this series was presented as a case abstract at the Iowa chapter of ACP on October 27, 2022.

## Case presentation

Case 1 

**Figure 1:**
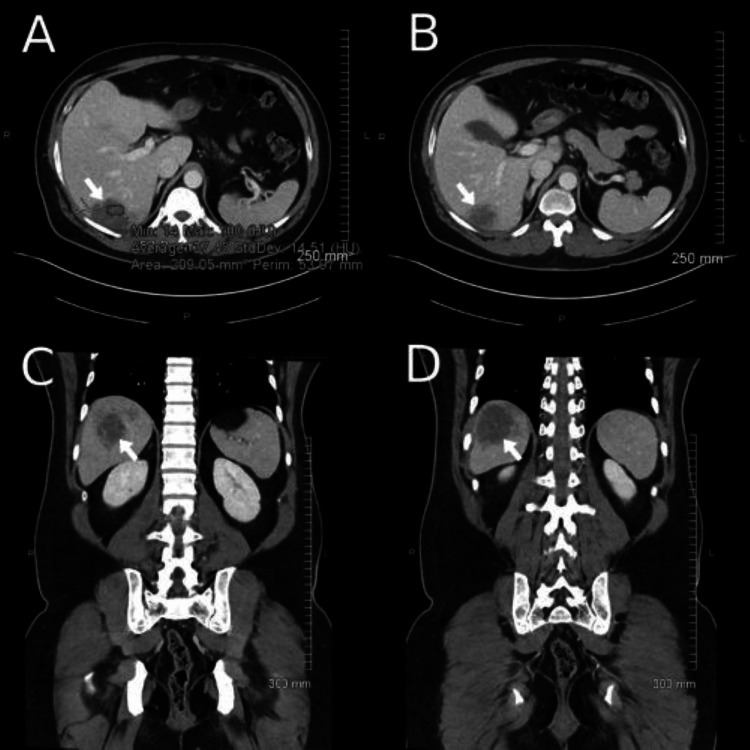
A-B Transverse slices from CT abd/pelvis demonstrating liver abscess. C-D Coronal slices from CT abd/pelvis demonstrating liver abscess.

A 52-year-old Malaysian male presented to the hospital with a chief complaint of right-sided abdominal pain which began nine days prior with associated fevers and chills. He had been sent to the emergency room from urgent care at that time due to concern for pneumonia. Vitals at this time showed a temperature of 100.2°F, heart rate of 124 bpm, and blood pressure of 148/99 mmHg. Labs at presentation were significant for leukocytosis of 19.2 K/mm3, elevated ALT at 92 international units per liter, elevated AST at 96 international units per liter, and elevated procalcitonin at 1.92 ng/mL. Due to abdominal pain, a computed tomography scan of the abdomen and pelvis was obtained which showed evidence of a benign-appearing liver lesion of approximately 5.7cm (Figure 1).

The patient was discharged on Nirmatrelvir/Ritonavir as he tested positive for COVID-19 infection. Blood cultures drawn from this emergency room visit grew* Klebsiella pneumoniae* within 24hrs. The patient was notified and instructed to return to the hospital. However, he delayed re-presentation by 8 days. On secondary evaluation, the patient stated that he had been having persistent right flank pain and intermittent fevers. Further history revealed that the patient had immigrated from Malaysia many decades prior and had visited five years prior to his presentation.

**Figure 2:**
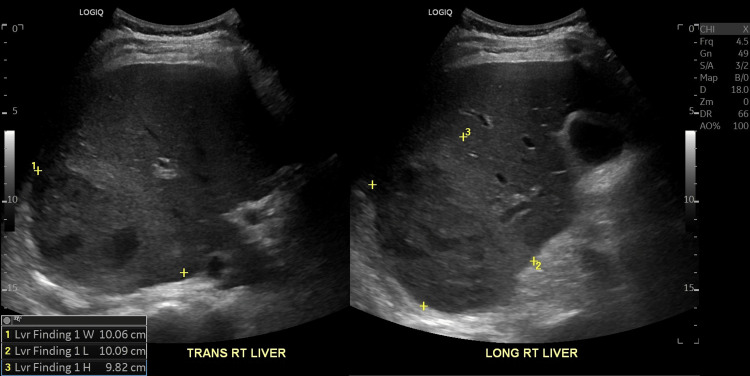
Ultrasound right upper quadrant demonstrating liver abscess.

On physical exam, the patient had jaundice and diffuse abdominal tenderness. Labs from re-presentation demonstrated a leukocytosis of 19.5 K/mm3, elevated ALT at 71 international units per liter, elevated AST at 46 international units per liter, elevated bilirubin at 1.8 mg/dL, and elevated procalcitonin at 5.51 ng/mL. An abdominal ultrasound showed a 10 cm hyperechoic focus seen in Figure 2.

**Figure 3:**
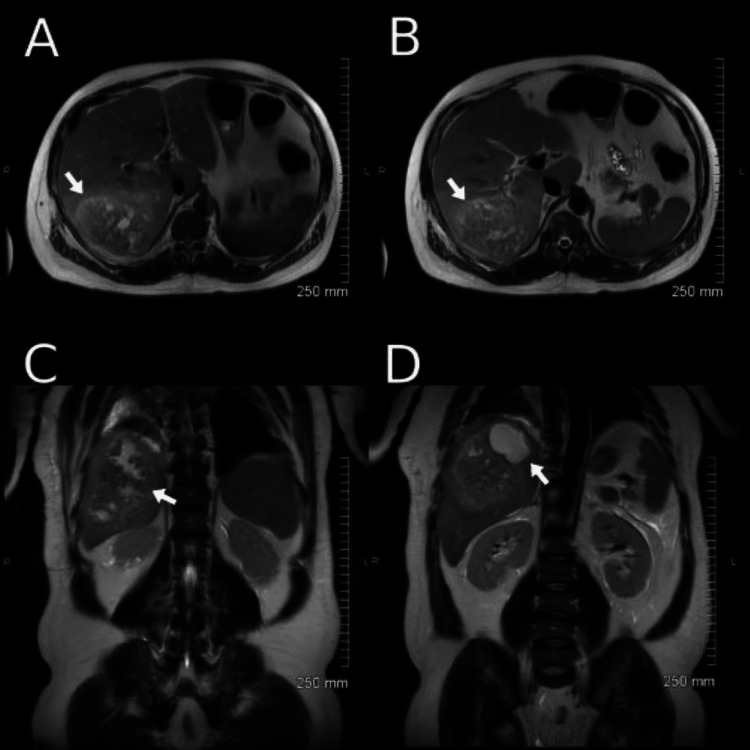
A-B: Transverse slices of MRI abd/pelvis further characterizing the liver abscess. C-D: Coronal slices of MRI abd/pelvis further characterizing the liver abscess.

After discussion with radiology, a follow-up abdominal magnetic resonance imaging was obtained which showed a 13cm multi-septated posterior right hepatic lobe abscess with segmental thrombosis of the right hepatic vein (Figure 3).

**Figure 4:**
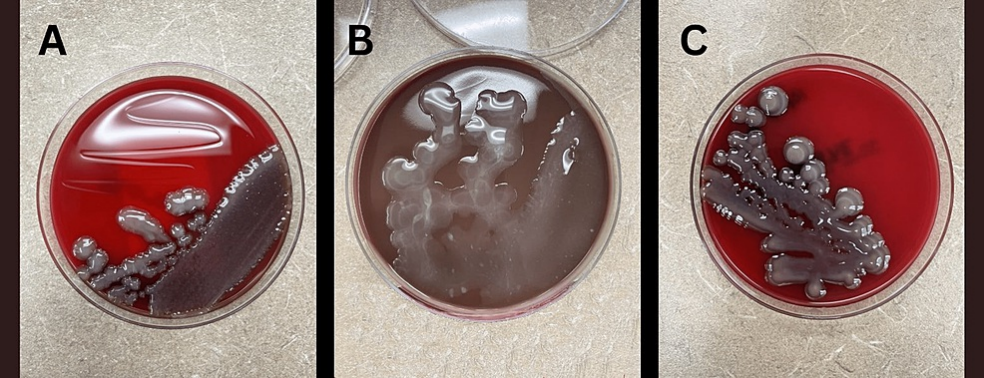
A: Blood culture growing on blood agar plate from Case 1 demonstrating mucoid colonies. B: Blood culture growing on chocolate agar plate from Case 1 demonstrating mucoid colonies. C: Blood culture growing on blood agar plate from Case 2 demonstrating mucoid colonies.

The patient was admitted for a pyogenic liver abscess with obstructive jaundice and *Klebsiella pneumoniae* bacteremia. A percutaneous drainage tube was placed on day 1 to obtain source control. A blood culture drawn from day 0 of hospitalization showed no growth. Culture from the liver abscess grew* Klebsiella pneumoniae* in mucoid colonies (Figure 4) which were resistant to ampicillin and sensitive to amoxicillin/clavulanate, ampicillin/sulbactam, cefazolin, cefepime, ceftazidime, ceftriaxone, ciprofloxacin, ertapenem, gentamicin, levofloxacin, meropenem, piperacillin/tazobactam, tobramycin, and trimethoprim/sulfamethoxazole. The patient was initially treated with piperacillin-tazobactam 4.5g q6hrs. After obtaining the culture data, therapy was narrowed to oral levofloxacin 750mg daily. A total antibiotic course of four weeks was planned. With therapy, the patient’s jaundice and abdominal pain improved as expected and had resolved by discharge on hospital day 5. Two-week follow-up noted improvement on imaging by a decrease in size, but incomplete resolution of the abscess which remained at 7.3 cm in greatest dimension. The drain was changed and the antibiotic course continued. After an additional two weeks, the drain was removed due to cessation of drainage, and imaging showed an interval reduction in size. At this time antibiotic therapy was changed to oral cefadroxil twice daily as the patient started to develop Achilles tendon pain. Repeat imaging performed two months later showed a complete resolution of the abscess. Therapy was extended four weeks from this imaging. The total duration of antibiotic therapy was 19 weeks from the first day of presentation.

Case 2 

**Figure 5:**
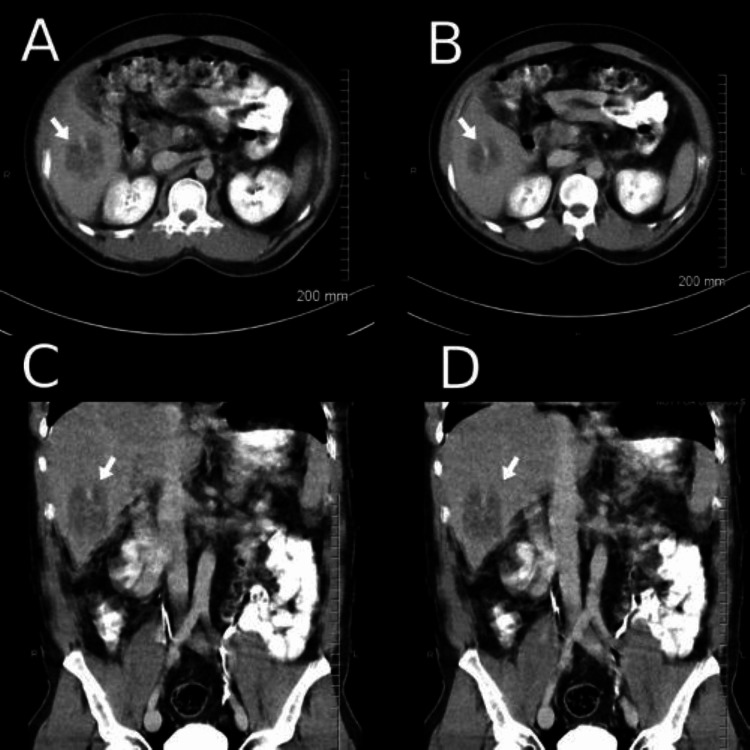
A-B: Transverse slices of CT abd/pelvis demonstrating liver abscess. C-D: Coronal slices of CT abd/pelvis demonstrating liver abscess

A 47-year-old man who emigrated from Thailand 11 years prior presented to the hospital with a chief complaint of fevers, chills, and body aches ongoing for seven days. He had an associated non-productive cough and generalized abdominal pain. He took no medications. The patient presented with a temperature of 98.5°F, a heart rate in sinus rhythm between 150 to 160 bpm, and a blood pressure of 84/62 mmHg. Labs from admission demonstrated leukocytosis of 13.8 K/mm3, elevated AST at 84 international units per liter, elevated ALT at 61 international units per liter, elevated bilirubin at 1.6 mg/dL, elevated procalcitonin at 3.11 ng/mL, and elevated CRP 29.8 mg/dL. The patient was diagnosed with septic shock and admitted to the intensive care unit for further care. Broad spectrum coverage was started with vancomycin 1000mg bolus dose followed by 750mg every 12 hours, metronidazole 500mg every 8 hours, and cefepime 2g every 12 hours. Due to elevated transaminase, an ultrasound of the abdomen was obtained which showed evidence of a 4.5cm hepatic lesion concerning abscess. However, the study was technically limited. CT with IV contrast demonstrated a 4.3cm right hepatic lobe abscess (Figure 5).

On hospital day 1, a drain was placed which yielded purulent material that was subsequently sent for culture. Blood cultures preliminarily identified gram-negative rods and vancomycin was discontinued. On hospital day 2, blood cultures grew* Klebsiella pneumoniae* resistant to ampicillin and sensitive to amoxicillin/clavulanate, ampicillin/sulbactam, cefazolin, cefepime, ceftazidime, ceftriaxone, ciprofloxacin, ertapenem, gentamicin, levofloxacin, meropenom, piperacillin/tazobactam, tobramycin, and trimethoprim/sulfamethoxazole. On hospital day 3, aspirate cultures grew *Klebsiella pneumoniae* confirming pyogenic liver abscess. Culture plates grew *Klebsiella* in mucoid colonies (Figure 4). Antibiotics were de-escalated to ceftriaxone 2 grams daily. The patient’s shock was resolved and he was transferred out of the intensive care unit. The patient’s care continued on the general medical floor until discharge on hospital day 6. He was transitioned to oral levofloxacin 750mg daily with instructions to follow up in seven days for evaluation of drains and to follow up with infectious disease for repeat imaging in two weeks to assess for resolution of the abscess. The drain was removed 17 days after discharge due to cessation of drainage. No additional drain was placed. Repeat imaging at five weeks showed a 3.2cm fluid collection at the abscess site. Therapy was extended and repeat imaging performed two weeks later showed a persistent abscess at 1.8cm. Therapy was changed from levofloxacin 750mg daily to cefadroxil 500mg twice daily to avoid complications of long-term fluoroquinolone use. The treatment course was planned to be extended with follow-up imaging eight weeks after the antibiotic change. However, the patient did not return for the follow-up appointment or imaging. The total duration of treatment up to this point has been nine weeks.

## Discussion

Hypermucoid strains of *Klebsiella* have been known in rare instances to cause pyogenic liver abscess due to translocation of gut bacteria without a history of predisposing hepato-biliary pathology [3][5][6]. We present two cases of *Klebsiella pneumoniae* liver abscesses. Both patients had a remote history of travel from Southeast Asia prior to immigration to the United States. Presumably, both individuals acquired these hypermucoid strains of Klebsiella that lead to colonization prior to immigration. Translocation of bacteria endemic to the individual's gut occurred in both cases, allowing for abscess formation and growth. The unique nature of no recent travel to Southeast Asia underscores the need for a higher degree of clinical suspicion for this pathology. Furthermore, neither individual possessed a history of hepato-biliary disease that would predispose them to infection. Providers should stay alert to the possibility of such infectious diseases as incidence rises outside of traditionally endemic regions. Definitive management requires source control or drainage of abscess and a prolonged oral antibiotic course for up to 4 to 6 weeks [5][7]. 

## Conclusions

Hypermucoid strains of Klebsiella are known to cause pyogenic liver abscesses. Prevalence is increased in Southeast Asia, which is a known endemic region. Providers should maintain a high degree of clinical suspicion for this etiology as prompt recognition of the disease may prevent progression to a more serious illness. Definitive management consists of source control via drainage of abscess and prolonged antibiotic course for up to 4 to 6 weeks. 
